# Distinct mechanisms and functions of episodic memory

**DOI:** 10.1098/rstb.2023.0411

**Published:** 2024-09-16

**Authors:** Sen Cheng

**Affiliations:** ^1^ Institute for Neural Computation Faculty of Computer Science, Ruhr University Bochum, Bochum 44780, Germany

**Keywords:** scenario construction, episodic memory traces, semantic information, mental time travel, hippocampus

## Abstract

The concept of episodic memory (EM) faces significant challenges by two claims: EM might not be a distinct memory system, and EM might be an epiphenomenon of a more general capacity for mental time travel (MTT). Nevertheless, the observations leading to these arguments do not preclude the existence of a mechanically and functionally distinct EM system. First, modular systems, like cognition, can have distinct subsystems that may not be distinguishable in the system’s final output. EM could be such a subsystem, even though its effects may be difficult to distinguish from those of other subsystems. Second, EM could have a distinct and consistent low-level function, which is used in diverse high-level functions such as MTT. This article introduces the scenario construction framework, proposing that EM crucially rests on memory traces containing the gist of an episodic experience. During retrieval, EM traces trigger the reconstruction of semantic representations, which were active during the remembered episode, and are further enriched with semantic information, to generate a scenario of the past experience. This conceptualization of EM is consistent with studies on the neural basis of EM and resolves the two challenges while retaining the key properties associated with EM.

This article is part of the theme issue ‘Elements of episodic memory: lessons from 40 years of research’.

## 1. Introduction

Tulving [1] coined the term *episodic memory* (EM) to describe a memory of a particular personally experienced event or episode. EM is a ubiquitous part of human experience and, therefore, an important subject in several scientific disciplines such as psychology, neuroscience and philosophy. However, despite decades of intense research efforts, it remains disputed how to operationalize EM. Many disparate experimental paradigms have been developed that are thought to be diagnostic tests of EM: from study-list learning over the what–where–when (WWW) paradigm to the autobiographical memory interview.

The classic paradigm for testing EM uses study lists, mostly consisting of words, and free recall [2,3]. Participants have to commit a given list of items to memory during the study phase and recall as many list items as possible during the test phase. The main advantages of this approach are that experiments are highly controlled and that memory performance can be quantified precisely. This basic paradigm has been used in many modified versions, e.g. survival processing [4,5], directed forgetting [6,7] and false memory [8,9].

Since free recall and other experimental paradigms are difficult, if not impossible, to implement in nonhuman animals and nonverbal humans, the WWW paradigm was proposed as an operationalization of EM [10,11]. Importantly, to qualify as EM, the memory system should, first, store the event information in such a way that WWW information can be retrieved separately, but, importantly, as conjunctions of WWW [12]. Second, this integrated representation should be encoded in a single experience. However, it has been argued that WWW information is neither necessary nor sufficient for EM [13].

A very different paradigm for testing EM in humans is the autobiographical memory interview. Participants are asked to recall significant events from their autobiography that typically occurred a few years or decades ago [14]. Sometimes the retrieved memories are corroborated by external sources such as diaries [15] or family members [16], but often the retrieved items are simply counted [17,18]. Unfortunately, there is no universal measure for autobiographical memory performance [19].

The lack of a standard experimental paradigm for studying EM is a symptom of a larger issue: there is no consensus on how to conceptualize EM. Here, I will focus on the two main challenges. The first challenge is distinguishing EM from semantic memory (SM), by which I mean the memory of facts^1^ [20]. This question has been much debated in the past, e.g. it was featured in a book 25 years ago [21], but the debate has not been resolved [22–24]. The problem is that the contents of EM and SM can be very similar. For instance, if one remembers seeing a toaster in a friend’s kitchen, it could be because we really saw it there (EM) or because one expects it to be there based on previous experiences in other kitchens (SM), even though the toaster was actually located in the bathroom during that particular visit. By placing objects in incongruent rooms, Zöllner *et al.* [25] dissociated retrieval of EM (where the object actually was) from retrieval of SM (where the object is usually found) and from guessing (unrelated third room). Task-irrelevant objects were less likely to be encoded into EM, and the subjects’ memory of their location more often reflected SM.

The second challenge is directed at whether EM is about memory at all. Retrieving memories of past events, the challengers claim, does not confer an evolutionary benefit and therefore could not have been selected for by evolution. Instead, they suggest that mental time travel (MTT) evolved to enable humans to plan for and secure future needs by simulating future events [26–29]. I will call these episodic simulation of future (ESF) events. Critically, the MTT view implies that EM is a byproduct of the MTT system misdirected towards the past, as for instance in this quote [27, p. 302]: ‘The fact that episodic memory is fragmentary and fragile suggests that its adaptiveness may derive less from its role as an accurate record of personal history than from providing a “vocabulary” from which to construct planned future events (and perhaps to embellish events of the past)’.

After briefly reviewing the two challenges in §2, I will suggest that EM is probably not a distinct memory system according to the hierarchical taxonomy of memory found in textbooks [30,31]. Instead, I will propose that a semantic system can be distinct from an episodic system, even if there is a large overlap between the two, as long as there is at least one component in one of the systems that is not found in the other and this component is critical for different functionality. Similarly, I will argue that EM and ESF are distinct systems. In §3, I will introduce the scenario construction framework with three key components: EM traces, semantic information, and scenario construction. I will use these components in §4 to make concrete how EM differs from SM, while overlapping to a large degree. In §5, I will dig deeper into the nature of EM traces, and how they might be modified post-encoding. This malleability of EM traces, in addition to the fact that EM traces are underdetermined, accounts for the generative nature of EM ([32–34], but see [35–37]). In §6, I will discuss how EM can change future behaviour in ways that do not require ESF. In §7, I will discuss the relationship between EM and MTT and conclude that, while they share common processes and information, they are distinct systems [38].

## 2. Is episodic memory a distinct memory system? two major challenges

Sherry & Schacter [39] suggested four criteria for judging whether memory systems are distinct from one another: the memory systems should (i) have distinct rules of operation, (ii) store different types of information, (iii) have distinct neural substrates, and (iv) be functionally incompatible. The first criterion comes in two versions: the strong version demands that the rules of operation do not overlap, while the weak view allows overlap, as long as there are components that are distinct to one system.

Those who argue that EM and SM are not separate memory systems might use these criteria to argue as follows. (i) EM and SM are both declarative memories (in contrast to procedural) [30]. (ii) The memory’s content is not different between EM and SM, it is solely the phenomenological experience during retrieval [40]. According to Klein, declarative memories accompanied by autonoetic consciousness^2^ are EM, whereas the same content accompanied with noetic consciousness would be SM. (iii) There are large overlaps in brain activation between EM and SM [41,42]. (iv) EM and SM are functionally compatible, even synergistic, as semantic information can be added during an EM retrieval (synergistic ecphory model) [26].

While Sherry & Schacter [39] developed their criteria to distinguish between memory systems, it seems that they could also be applied to evaluate whether the systems underlying EM and ESF are separate systems. The proponents of MTT ‘might’ argue that the criteria are all violated. (i) Both EM and ESF are supported by the same rules of operation, namely scene construction in the hippocampus [43,44]. (ii) Episodic and semantic information fuel both EM and ESF [27]. (iii) There is a massive overlap in brain activation between EM and ESF [28]. (iv) Memory of past events (EM) has no function other than ESF [27].

**Figure 1.**
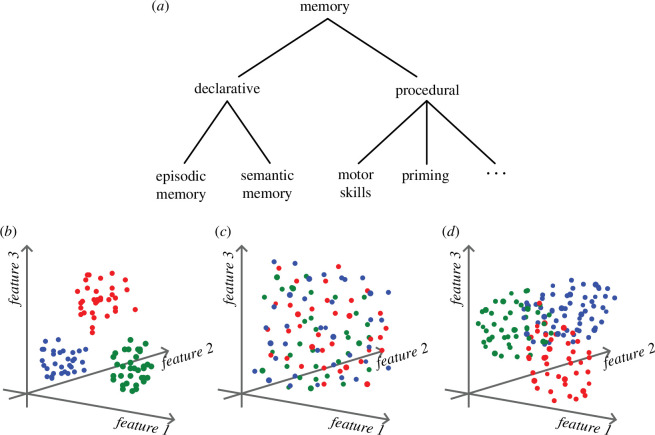
Different organizational principles applied to memory systems. (*a*) Hierarchical taxonomy, (*b*) distinct clusters, (*c*) indistinct arrangement, (*d*) continuum of properties.

We suggest that there is a fundamental issue with the two research programmes that has been largely overlooked. All statements about distinctness are relative to how large a difference one is willing to accept when calling two things the same. Let us call this level the tolerance threshold. Except in highly artificial systems, no two items are exactly identical, so the tolerance threshold cannot be zero for cognitive entities. The tolerance threshold varies between researchers, is almost always chosen implicitly, and often shifts depending on the context. One crucial factor that determines the tolerance threshold is the conceptual model that is adopted by the researcher [45,46]. If that model is a hierarchical taxonomy of memory (figure 1*a*), the tolerance threshold is very low. In particular, it requires that one should be able to show a double dissociation for two memory systems to demonstrate functional incompatibility [39]. If that is the bar, one would think both challenges are successful and EM is probably not distinct from SM or ESF. However, I believe that the time has come to move beyond the model of hierarchical memory systems and adopt a more nuanced model for the relationship between EM, SM and ESF. Rubin [47] has suggested a three-dimensional space in which EM, SM and other types of memory can be embedded.

I build on this idea and place not memory systems in a multidimensional space, but individual memories and episodic simulations. Each memory or simulation is represented by a datapoint that is placed in the multidimensional space according to its properties, which are measured along the dimensions of memory features (figure 1*b–d*). The fact that instances of EM, SM and ESF can be placed into the same multidimensional space does not say anything about whether the three are distinct or not. What matters is how instances of EM, SM and ESF are distributed in this multidimensional space. If the datapoints form three identifiable clusters (figure 1*b*) that correspond to our intuitive notion of EM, SM and ESF, then the three systems are distinct. If, by contrast, the datapoints are evenly intermixed, then they are clearly not the product of distinct systems (figure 1*c*). Finally, the datapoints could form a continuum where on one pole there is only EM, at another pole only SM, and at a third pole only ESF, and in between a mixture of the three (figure 1*d*). This case is most difficult to interpret since the datapoints could be produced by one system that is controlled by one or more continuous parameters, or three distinct systems that are mixed in different proportions. Although there are currently no experimental studies of this kind, it would be possible to implement them. For instance, a possible study could collect detailed reports of past episodes, fact knowledge and future simulations; score these reports along many different dimensions; use some kind of factor analysis; and then place each report in the space of the emerging factors.

The outcome of such an experiment is unlikely to look like the first or second case and more likely to look like the third case, possibly even less clear. In the following, I suggest that while EM, SM and ESF have overlapping features, each has unique properties that distinguish it from the other two. In particular, EM is a memory system in its own right distinct from ESF and SM. The interpretation of experimental studies will critically depend on the conceptual model of the relationship between EM, SM and ESF. Here, I focus on EM and hence develop a theoretical framework for EM.

## 3. The scenario construction framework

**Figure 2.**
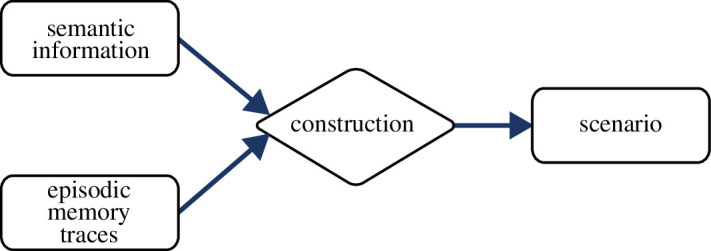
Scenario construction framework. Episodic memory traces, which contain the gist of an episode, are enriched with semantic information to construct a scenario.

Here, I propose the scenario construction framework [48] and discuss in the remainder of this article how it clarifies the relationship between EM, SM and ESF. This framework suggests that EM references one particular experienced episode, stores only the gist^3^ of the sequence of events in that episode, and does not *a priori* involve autonoetic consciousness during retrieval [13,48,55–58]. According to this view, three main components are involved in EM: EM traces, semantic information and scenarios. These components interact strongly during memory encoding, consolidation, updating and retrieval (figure 2).

In my framework, EM traces^4^ encode the essence of EM, i.e. the gist of the sequence of events. In this framework, ‘gist’ does not represent a complete record of the detailed and rich experience of the past and is underdetermined in two aspects. First, during the experience of an episode, not everything is attended to or encoded [61]. After encoding, some information might be lost in storage [62,63]. In addition, some episodic information might be temporarily inhibited during retrieval and not accessible [64–66]. Second, episodic information is represented at the most abstract level that is relevant in that one episode [49–51]. The relevant level is established dynamically for each episode, depending on the context and attention during encoding [67], and may be quite detailed in some cases and abstract in others. Experimental evidence in levels of processing [68], and false memory in the Deese–Roediger–McDermott paradigm [8,9], lead us to believe that the most relevant level is often at a rather abstract level such as object categories or narratives. However, the episodic gist can include lower perceptual information and be encoded at multiple levels simultaneously.

The storage of EM trace critically depends on the hippocampus, no matter how remote the memory [13,56]. I have suggested previously that an episode in EM consists of a sequence of events [13] and that EM exploits temporal sequences of activity patterns as a format of representation [55,69].

*Semantic information* ^5^ frequently consists of general information about the world and oneself, but can refer to particular facts. I view semantic information as (statistical) regularities of features occurring or co-occurring in the world. Semantic information either was extracted from multiple experiences or applies to more than one instance [20], is mostly categorical, and refers to prototypical properties of objects or people and the relationships between them [1,70]. However, it can also encompass idiosyncratic features and relationships, as long as they are consistent across different episodes, such as, for instance, the name and location of a particular building. Semantic information can be encoded at different abstraction levels, including that of low-level sensory representations, and does not necessarily have to be conceptual, nor does it have to involve schemas [35,71,72], even though it could do so.

Unlike EM traces, semantic information does not depend on a temporally structured format of representation. However, semantic information might include temporal or sequential information, which are called scripts [73–75]. They refer to a class of episodes, i.e. they describe how events generally unfold in a certain type of episode, such as a visit to a restaurant. By contrast, an EM trace references a particular episode, e.g. ‘Last Friday evening I ate mushroom pizza at my favourite restaurant together with my spouse’. The neural substrates also differ. While storage of EM traces requires the hippocampus, script knowledge does not [76]. Script knowledge has been associated with the prefrontal cortex [77,78]. Representation of the more abstract type of semantic information appeared to be centred around the anterior temporal lobes [79,80]. Generally, the representation of semantic information is distributed across the cortex [81,82].

Scenarios are extended in space and/or time and contain information about sequences of events that involve a setting, participants and interactions [48,83]. One is able to explore a scenario by, e.g. mentally moving through space, focusing on different participants and taking different perspectives [84,85], and/or moving through the temporal sequence of events. Thus, scenarios can be thought of as mental simulations of an episode. Our notion of scenario subsumes the scene proposed by Maguire and colleagues as a coherent spatial organization constructed during memory recall and other cognitive functions [43,44]. In addition to this spatial arrangement, scenarios are also extended and arranged in the temporal domain. Scenarios are constructed when EMs are retrieved, when ESFs are generated, and to represent the current world around us. Scenario construction does not require a deliberate effort and can occur automatically [86].

During an episodic experience, cortical representations are activated by sensory inputs as well as top-down processing. Attentional processes, task demands and bottom-up processes select the appropriate level of abstraction and spatial region that make up the episodic gist. During encoding, an EM trace is generated such that the neocortical representation of the gist of the episode can be reactivated at a later point in time. Retrieval is triggered by an external or internal cue, which selects an EM trace. I do not, at this point, commit to a particular model of how memories are selected for retrieval. This could be accomplished, for example, based on a simple pattern completion operation or more elaborately as in the model of Conway & Pleydell-Pearce [87]. During retrieval, a scenario is constructed that contains an approximation of the episode referenced by the retrieved EM trace. Elements and details, which were not attended to, can be added during scenario construction based on semantic information (figure 2).

The construction of scenarios of the past is a feature that our framework shares with the simulationist account [88], which argues that a reliable simulation system is sufficient and denies the need and reality of an EM trace. The simulationist account raises the question why my framework insists on some contribution of a causal trace to an EM given that I am willing to concede that much of EM is constructed during retrieval. Werning [89] has argued that causal EM traces must be present to ensure that simulations are reliably about a past experience. This begs the question how much of the information in a memory needs to be contributed by the trace for the memory to count as EM. I believe that no specific fraction can be given, but rather that the criterion should be whether the episode referenced by a memory is identified uniquely with a high probability (e.g. [37]).

In the following sections, I will further flesh out the framework in four corollaries and discuss the supporting evidence.

## 4. The interaction between semantic information and episodic memory traces

The serial–parallel–independent (SPI) model [90,91] suggests that the relationship between SM and EM is process- dependent. During encoding, sensory information passes through the semantic system before it is encoded into EM. The information is stored in different memory systems in parallel, and retrieved independently from each system. While the SPI model accounts for many EM phenomena [90,91], it does not readily explain why semantic learning in developmental amnesics is significantly slowed down [92] or why patients with semantic dementia develop deficits in EM recall as the disease progresses [93,94]. I suggest that a stronger integration of episodic and semantic information is required to account for the large diversity of experimental findings on EM versus SM.

### (a) Corollary 1

**Figure 3.**
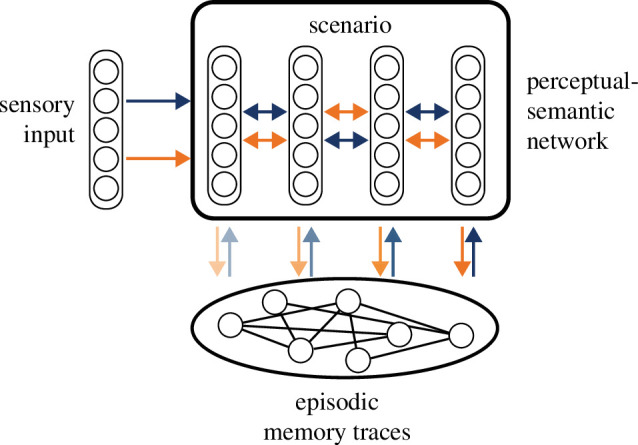
Network model of interaction between episodic memory traces and the perceptual–semantic network. Orange and blue arrows indicate information flow during encoding and retrieval, respectively. The fading indicates the decreasing likelihood of interactions. Lateral connections within the neocortical layers are omitted for clarity.

I propose that semantic information is represented in a hierarchical neural network (figure 3), much like the visual system [95] and deep neural networks in machine learning [96]. In this network, neurons are arranged in layers consisting of units with similar response properties. Different layers are connected to each other via feedforward and feedback connections. Lateral recurrent connections within layers enable local processing such as noise reduction and pattern completion. When fed with sensory inputs, I hypothesize that each layer represents increasingly abstract features, eventually giving rise to object categories and relationships between them. Owing to the lack of a clear dividing line between the perceptual and semantic representations, I call this network the perceptual–semantic network. Importantly, owing to capacity limitations, EM cannot store the complete activation pattern of the perceptual–semantic network for all experiences. Neither would that necessarily be desirable since events rarely, if ever, repeat in exactly the same way, and compressing the information encoded in EM can facilitate the generalization of EM to other novel situations. Therefore, attention selects which layer(s) (the level of abstraction) and which units (the spatial region) to focus on. The partial activation pattern (the gist) is then encoded in EM traces after a single exposure. As hypothesized in trace minimalism [89], these highly impoverished EM traces do not have representational contents by themselves. They give rise to EM only in combination with the perceptual–semantic network [97] and scenario construction.

### (b) Discussion of Corollary 1

The strong integration of the components involved in EM in the scenario construction framework accounts for the activation of diverse brain regions during EM retrieval [81], while at the same time assigning specific roles to the neocortex and hippocampus. I suggest that the neocortex represents more stable semantic information, while the hippocampus encodes idiosyncratic event-specific episodic information. As a result, plasticity in the hippocampus is faster than in the neocortex, as suggested by the two-stage model [98] and complementary learning systems theory [99,100].

Modelling the neocortex as a deep neural network has attracted much attention and accounts for different representational formats along the neocortical processing hierarchy [101,102]. The initial models were feedforward networks that process information sequentially layer by layer. Such sequential propagation of neural activity through the cortical layers during encoding has been observed experimentally [103]. However, more recently it was shown that recurrent neural networks (RNNs) accounted for more variance in the data than feedforward networks could [104]. It therefore appears that the best model for the neocortex might be a recurrent network with strong feedforward connectivity between layers, which is what I adopt here.

This framework predicts that reactivating neocortical representations during retrieval requires bi-directional interactions between the hippocampus and neocortex. During encoding, first the cortex is activated and then the hippocampus. During retrieval, the cue leads to an activation of the cortex first, which then triggers the activation of EM trace in the hippocampus, which in turn leads to a reinstatement of the cortical representations similar to those that were active during encoding. New methodological developments using representational similarity analysis of fMRI data make it possible to study the representational formats of memory representations, and the hypothesized order of activation during encoding and retrieval has indeed been observed [103].

If there is even a weak topography in the connections between neocortex and hippocampus, then the subregions of the hippocampus would inherit the properties of the hierarchical neocortex. In other words, there would be a gradient of abstractness or length scale from anterior to posterior hippocampus.

The point of EM is to rapidly encode episodic information efficiently and retrieve it robustly. This requires compression and reconstruction. Our framework hence suggests that EM is strongly dependent on the semantic system, as observed experimentally [105]. EM recall is more accurate when it is based on a mix of episodic information and prior knowledge [106]. Semantic information about the temporal structure of episodes, such as narrative structure [107,108] or scripts, can affect scenario construction, particularly when the EM trace is incomplete. A computational model by Fayyaz *et al.* [97], based on the scenario construction framework, performs semantic completion of incomplete EM traces and reproduces the experimental observations of Zöllner *et al.* [25] mentioned in §1. Intriguingly, the model produces graded interactions between episodic and semantic information, even though the two systems are clearly separated in the model.

Finally, I need to explain what I mean by the statement that EM traces have no representational contents by themselves. This might seem contradictory since, in my framework, EM traces must reference a particular episode. So how could they do so without having any representational content themselves? Since EM traces point to semantic representations, they can be linked to the contents of the targets they point to and hence the traces can reference an episode by pointing to the right semantic representations that were active during the experience. Nevertheless, the traces themselves remain pointers and do not become representations, nor do they acquire representational contents. This distinction is important to account for cases where either semantic representations or EM traces change after encoding, but the other one does not. If semantic representations change while pointers in the EM trace remain constant, the pointers will reference something different. In that case, I predict that the content of the EM is modified and might become inaccurate.

## 5. Episodic memory traces and their post-encoding dynamics

Most discussions of memory traces have explicitly or implicitly assumed that, to serve their function in establishing an ontological and causal link to the initial experience, EM traces have to be fairly stable once they are encoded. Stable memory traces are most clearly articulated in the engram theory [109], but also feature prominently in the multiple trace theory [110]. This view has difficulty in accounting for the generativity of EM and is at odds with large-scale dynamics of hippocampal representations in stable environments ([111,112], but see [113]). The extreme opposing view would be simulationism, which holds that no causal EM traces are required for EM [28,88]. However, simulationism does not account for the empirical reliability of EM [83]. It appears that neither extreme position is able to account for the full range of observations in EM, and an intermediate position is required, one in which EM retains sufficient information about a past episode so as to provide a unique reference to it, and at the same time allows modifications to the memory and generativity.

### (a) Corollary 2

I hypothesize that EM traces establish an ontological and causal link to a particular episode in the past. EM traces are sequential in nature, and the hippocampal circuit is optimized for instant storage of neural sequences [55]. Each element in the EM trace acts as index or pointer to cortical representations [114–116], i.e. EM traces by themselves do not represent external variables. So, when I say that EM traces are activated or discuss the contents of EM traces, I refer to the neocortical representations that the traces point to and that are, hence, linked to the EM trace. EM traces are highly dynamic, and changes in EM traces introduce changes in EM content. Post-encoding modifications of EM traces could be driven by passive changes such as decay, random drift, or interference by EM traces encoded later. EM traces can also be changed actively, for instance, when the level of abstraction in EM changes over time [52,63]. In other cases, EM traces are updated to keep them consistent with each other and with the semantic network—a process which Káli & Dayan [117] hypothesize is driven by memory replay, or when a novel experience is similar to a previously encoded one. In the latter case, either the previous EM traces is updated or a new EM trace is linked to the same original episode.

### (b) Discussion of Corollary 2

Trace minimalism posits that memory traces are causal links to the experienced episode, but contain only minimal information about the episode [89]. This view accounts for both the reliability and generativity of EM and also allows changes to the EM traces after encoding. A rather loose binding between EM traces and representations in the perceptual–semantic network (EM traces store pointers) lends further support to trace minimalism since it allows either semantic information or EM traces to change without necessarily imposing a change on the other. However, this introduces a new challenge. If semantic representations or information changes after EM traces have been encoded, the established traces will activate those new representations, which will lead to distortions of the EM [117]. EM distortions could also result if EM traces change post-encoding.

Post-encoding modifications of EM are common [118] and even strong memories, so-called flashbulb memories, are no more immune to modifications than everyday EM [119]. Post-encoding modifications can be induced by information that is provided after the event [120], the so-called misinformation effect [121]. Most dramatically, false autobiographical memories can be implanted by suggestion [122] or through forced confabulation [123]. The malleability of EM traces is consistent with the considerable evidence suggesting that similar episodes that are experienced later are accepted by subjects as the original [124–127]. Since the original is recognized as having been experienced as well, it suggests that the original EM trace was altered by the new experience. However, it remains an open question whether the original EM trace was modified to encompass the new experience or two independent EM traces were linked to the same episode. Nevertheless, neuroimaging suggests that neural representations of the two versions remain distinct, even if subjects do not distinguish them at the behavioural level.

The flip side of the weak coupling between EM traces and semantic information is a relatively stronger coupling within the hippocampus and within the neocortex. Consistent with this view, I have suggested previously that the hippocampal circuit is specialized for rapid storage of EM traces by providing a pool of neuronal sequences in CA3, onto which external inputs are mapped via superficial entorhinal cortex [55,128,129]. This contrasts with theories in which sequences are imposed onto the hippocampal network by the neocortex, and plasticity then encodes these patterns in the weights of the hippocampal circuit [130,131] and suggestions that the sequential ordering is generated by prefrontal cortex [132]. The intrinsic sequence view also accounts for various experimental reports where replay sequences do not correlate with past experience [133–135] or occur before the experience has taken place, i.e. preplay ([136,137], but see [138]).

An interesting alternative view is that the hippocampus is required for scene construction, which underlies EM as well as ESF [43,44,139]. Counterarguments to this view are that amnesics show spatial reasoning skills [140,141] and can imagine future events [142], even if the amnesics’ performance differs from that of controls. The deficits of amnesics might be mainly in the phenomenology of the recollection, which can be easily manipulated by a simple induction technique [143], and measuring phenomenological experience using verbal reports is unreliable [144]. The fact that developmental amnesics do not suffer a deficit in scene construction is further evidence that scene construction can be learned independently of EM [145] and of the hippocampus [146].

## 6. Using episodic memory to drive learning and inference

Consider a function X that is hypothesized to involve EM, but also draws on other cognitive processes. Even though these other cognitive processes might have a significant effect on X, nevertheless X and EM are often taken to be synonymous. For instance, nonhuman animals might possess EM and employ it to solve the WWW task [10], but this does not necessarily mean that EM *is* WWW memory, or *vice versa*, and the WWW task might be only one of myriad ways of testing EM. On the other hand, there are claims that all the phenomenology that is associated with MTT are necessary, or even defining, characteristics of EM [26,147]. As a field, we need to more clearly distinguish between EM and a function X that draws on EM and acknowledge that the contribution of EM to X might be delayed and diluted.

### (a) Corollary 3

I hypothesize that EM provides access to information from past experiences for later inference and learning. I distinguish three modes. First, inference is the process of extracting information from memory that was not explicitly encoded [70], e.g. transitive inference [148,149]. Second, one-shot learning is the process by which the behavioural disposition is changed after a single experience [150]. Third, replay learning is a process by which a behaviour is acquired incrementally by repeatedly replaying multiple experiences that were stored in a memory buffer [151].

We distinguish between the EM that represents a past experience and the act of learning behaviour based on EM. For instance, EM traces are always encoded after single experiences, regardless of whether they later drive one-shot learning, incremental learning, or inference. It is even possible that the same EM trace is used for one-shot learning a certain behaviour, replay learning of another, and inference in a third instance. Furthermore, the three modes are not mutually exclusive, e.g. during one-shot and replay learning, new behaviours can emerge that were not previously executed in exactly the same sequence, which is inference. Also, a given behaviour could be the result of iterative improvement through multiple instances of learning and inference. Finally, one-shot learning might involve (a single) replay of an experience.

### (b) Discussion of Corollary 3

There is sometimes confusion between encoding EM and behavioural changes after a single experience, which is expressed in this quote [152, p. 8]: ‘the induction of the episodic-like memory must be per definition a one-trial learning event.’ According to our view, successful performance on the WWW task (as it is usually administered) is a form of one-trial learning, but encoding an EM by itself is not. One-shot learning requires, by definition, information that was encoded in a single trial, which is usually EM—but not always [153–155]. However, learning requires other processes that change the behavioural disposition based on the information in EM. The distinction between one-shot learning and EM is also important because information from multiple EMs, which are each stored in a one-shot fashion, might combine to drive changes in behaviour, e.g. during replay learning or inference, which are incremental.

I emphasize this point for two reasons. First, many learning processes that employ EM might be of the incremental type, such as acquiring semantic knowledge [56,156], skill learning [157,158] and even sensory learning [159]. These processes are far slower than the timescale of EM encoding and are not commonly associated with EM. Second, even if EM facilitates a particular learning process, learning might still be possible in the absence of EM because the learning ifself is implemented elsewhere. For instance, the neocortical network supports rapid plasticity in certain situations [160]. This view might account for why many learning tasks that are affected by hippocampal lesions can nevertheless still be learned without a hippocampus. Examples of this are fast mapping in amnesics [155], whereby the meaning of the word is directly integrated into cortical networks [161], and an amnesic actor who can learn new lines [162]. However, learning in these cases appears to be less efficient, e.g. developmental amnesics acquire novel semantic information more slowly and they require more repetition [163–165], and perhaps only recognition, but not recall, of semantic information can be acquired in adult-onset amnesia [166,167].

The extraction of semantic information supported by replay learning might be what occurs during systems consolidation [56]. This view of systems consolidation is consistent with complementary learning systems [99] and the two-stage model [98]. Both theories and our view distinguish between a fast-learning hippocampal system and a slow-learning neocortical system, but they differ on how information is represented in the two systems and how the systems interact.

Machine learning algorithms provide a powerful tool with which we can study whether and how EM facilitates learning in different tasks [168]. We have used reinforcement learning models of spatial navigation to show that EM could play a key role in facilitating spatial learning [169,170]. In particular, we have demonstrated that there are key differences between using EM in one-shot learning and in replay learning [150]. One-shot learning tends to learn in a greedy fashion and improves rapidly at the beginning, at the expense of reaching a suboptimal asymptotic performance. By contrast, replay learning is incremental and therefore slower initially, but reaches a near-optimal asymptotic performance.

Note that in these studies cited here, learning and inference are only possible because the agent stored specific experiences of the past and does not require a simulation of the future. Non-specific summaries of experiences or confabulated information lack the required information. For instance, to find your car after work efficiently, you have to remember where exactly you parked it earlier in the day. It is less helpful if you only know where you generally park your car around your workplace—unless, of course, you have a reserved parking spot—or if you imagine where you could have parked your car.

## 7. Episodic memory and its relation to mental time travel

It has been suggested that past experiences are stored in EM to make predictions of future events [27,28,171–174] and that autonoetic consciousness is required for MTT [26]. Here, I view MTT as the mental simulation, elaboration and exploration of a past or future episode. However, it is not clear how specific memories of the past are useful for ensuring future survival and reproduction. After all, the past does not repeat. It is also not clear why autonoetic consciousness should play such a central role in planning for the future. While this requirement makes it difficult, at a practical level, to study MTT in nonhuman animals [11,175] and nonverbal humans [176], my concern runs deeper. As I argued in §6, there might be many instances of inference and learning based on EM that are not easily interpreted as MTT. Therefore, there is no reason to single out MTT and autonoetic consciousness as special functions of EM. So, what is the relationship between EM, ESF and autonoetic consciousness?

### (a) Corollary 4

ESF is the result of scenario construction in the service of inference directed towards the future, but does not reference one particular past episode. (i) ESF can combine semantic information with EM traces from multiple different episodes, but does not necessarily have to involve any EM traces. If we conceptualize semantic information to also include information that is specific to oneself and/or single events—after all there are many regularities about singular items like oneself and specific events—then semantic information suffices to simulate a future event. This stands in contrast to conceptualizations of semantic information as general world knowledge. (ii) EM can be involved in episodic foresight in the absence of autonoetic consciousness, and (iii) the function of EM is not limited to ESF—see Corollary 3. Hence, our concept of EM is consistent with it being used in ESF, but rejects the view that EM is an epiphenomenon of ESF.

### (b) Discussion of Corollary 4

To be clear, the novel claim is not that imagination of the future is possible without EM traces. For example, Suddendorf & Corballis [27] already distinguish between future-oriented cognition based on the semantic and episodic systems, and evidence suggests that semantic future thinking is independent of the hippocampus [177]. Irish [178] suggests that semantic imagination occurs for imagination of temporally distant events or de novo. However, she suggests that semantic imagination is more schematic than episodic imagination, which occurs for temporally close events and is more personal. Rubin & Umanath [44] have suggested that, even if EM traces are involved, scenarios of the future are generated mostly using semantic information. For instance, simulating a future episode (e.g. the next family gathering at Christmas that I am going to host) benefits from the memory of a specific past episode (e.g. last year’s Christmas party), which might contribute some piece of information (e.g. my youngest nephew choking on a nut), so that I can prepare for the future episode (e.g. not serving nuts). However, it is reasonable that most of the scenario, and perhaps all of it, is based on general knowledge about the elements of the episode (e.g. family, homes and Christmas traditions) and specific information about the elements in the specific episode (e.g. my family, my home and my family’s traditions).

One could concede that there are two different types of ESF, one based purely on semantic information and another one also involving EM traces, similarly to how I argued in the case of the distinction between semantic information and EM. However, there is a key difference between ESF and EM. In the case of EM, the trace provides a reference/link to a particular episode that is being recalled, which makes it very different from confabulating a past episode, both phenomenologically and ontologically. In the case of ESF, there are only simulations of a hypothetical episode and, as far as I can see, it makes little difference what sources were tapped for the information that is being used to generate the scenario. Furthermore, EM is about episodes that have actually occurred, so they have a different epistemic status from ESF, which probably will not come about as imagined—except perhaps for highly artificial manmade situations. So, our novel claim is that ESF events can be generated without any involvement of EM traces, a view that might become more common [24,179].

My other suggestion that episodic foresight is possible without autonoetic consciousness might seem radical, but the same has been suggested for EM [180]. Henke and colleagues have shown that subjects can form memories of one-time events that share many features with EM, even though subjects are not consciously aware of these memories. These implicit memories can support complex inference [149,181], which is usually associated only with explicit memory. So, unlike the textbook taxonomy of memory suggests [30], explicit and implicit do not neatly map onto declarative and procedural memory, or its subdivisions. Nevertheless, there are differences. The explicit system might have higher cognitive flexibility and a lower memory capacity than the implicit system [182]. It therefore does not seem far-fetched to suggest that ESF is possible without conscious awareness, but this hypothesis awaits experimental confirmation. It also remains to be shown what differences, if any, there are between conscious and nonconscious generation of ESF.

The large overlap between neural activation patterns in ESF and EM recall does not rule out that they are distinct processes. The neural overlap might be due to the common neural mechanisms underlying scenario construction, semantic information [183] and EM traces. The difference between EM and ESF is that EM has to involve EM traces, suggesting that there should be a difference in activation of the hippocampus, where I suggest that EM traces are stored. Alas, many studies have not found such a difference between ESF and EM recall; however, questions remain about the methodology used in these studies [144]. I suggest that the temporal and spatial resolution of these studies was insufficient to resolve why the hippocampus was activated during ESF. Possibly, some EM traces were retrieved from the hippocampus to construct the scenario (however, this is not a prerequisite), EM traces about the current task were encoded in the hippocampus, or a combination of the two.

A final question is how the brain keeps the different operations of ESF and EM apart. As important as this question is, there is a far greater problem that the brain solves without issue: we can easily distinguish between a scenario that we are constructing (regardless of past- or future-orientation) and our perception of the world that is currently around us. As long as our brain can accomplish this, perhaps by source monitoring [28,184] or reality monitoring [185], to keep track of whether the constructed scenario is in the past or future appears straight forward.

## 8. Conclusions

On the basis of the ideas and evidence reviewed in the preceding sections, I now summarize the counterarguments to the two key challenges that I introduced at the beginning of this article.

### (a) Are episodic and semantic memory distinct memory systems?

According to the four criteria for distinct memory systems of Sherry & Schacter [39], I conclude that EM and SM are distinct memory systems.

— Figure 2 illustrates the distinct rules of operation. The strong view obviously does not apply since there is much overlap between the two systems. SM encodes, stores and retrieves semantic information. EM operates on semantic representations and relies on semantic information. However, EM traces and the interaction between neocortex and the hippocampus clearly differentiate the rules of operation between EM and SM. This corresponds to the situation shown in figure 1*d*. So, the weak view, according to which the memory systems might overlap as long as there are distinct components, applies.
— Regarding the informational content, I suggest to distinguish between episodic and semantic information not based on their content, but rather on the combination of how they were acquired (single experience vs repeated) and the flexibility of their representational format (sequential vs unconstraint). This is similar to the view of processing modes by [180].
— There are clearly distinct neural substrates underlying EM and SM, even if there is much overlap. Neocortex is the key substrate for semantic information and the hippocampus for EM traces.
— The two systems are functionally incompatible. The perceptual–semantic network alone cannot be used to encode EM, and the distinct component of EM, the traces, cannot be used to encode semantic information.

### (b) Is episodic memory a system distinct from episodic future simulation?

We also conclude that EM is a system distinct from ESF according to the same criteria.

— The strong view does not apply since there is much overlap between the two systems, e.g. scenario construction, and the use of semantic and episodic information, but there are clearly distinct rules of operation. In the case of EM, there are storage, consolidation and retrieval—processes that do not play a role for ESF^6^. Again, this corresponds to the situation shown in figure 1*d*.
— ESF does not need to, but could, use EM traces. By contrast, EM must be based on EM traces that are causally linked to a particular past episode. Therefore, there is a difference in information.
— The most reliable difference in neural substrates is probably in the source monitoring or reality monitoring network. However, there should generally be more engagement of the hippocampus in EM than in ESF, and stronger interactions between the hippocampus and the neocortex in EM.
— There is clear functional incompatibility. EM drives learning, whereas ESF drives planning. Even though both lead to adaptive changes in current and future behaviour, learning is a permanent change in disposition and planning is a temporary process.

### (c) Distinguishing features and predictions of the scenario construction framework

I conclude with a brief discussion of what separates the framework proposed here from the numerous previous theories about EM. I see one overarching difference. While many individual aspects of the framework have been suggested before (two complementary systems, pointers, multiple memory traces, scenario/scene construction, hierarchical semantic representations, etc.), the combination of these aspects is novel. The framework is integrative and coherent across different levels of description (neural mechanisms, cognitive processes and function) and across different memory phases (encoding, consolidation and retrieval). Furthermore, it integrates these two dimensions. It is this integrative nature that allows us to generate novel insights that could be tested in experiments. One example is that the framework provides a mechanistic explanation for the generativity of EM at the behavioural level. It is the split of episodic information between the pointer to the gist in the hippocampus and semantic information in neocortex. The impact on generativity of EM could be tested by transiently disrupting recurrent connections within neocortex or the connections between hippocampus and cortex during EM retrieval. Another example is that, in the framework, EM is always dependent on semantic information throughout all memory phases: encoding, consolidation and retrieval. This predicts, for instance, that manipulating semantic information after the encoding of EM would alter the contents of EM accordingly. Furthermore, the framework describes what happens during consolidation as semantic learning driven by replay of hippocampal EM traces, rather than transfer or transformation of equivalent information from hippocampus to cortex. The difference between the two views has implications for the observed behaviour after consolidation. In the former view, the behaviour will be different, more optimized, while in the latter view, performance will be similar or worse. This difference is expected to be subtle and we probably cannot use the performance on the experimental task as a measure since that is unlikely to be the objective of semantic learning. Finally, ESF in the framework uses the neural machinery of scenario construction, but differs from EM in that ESF does not reference one particular past episode and involves higher generativity and more semantic information than EM. Hence, activation of hippocampus is predicted to be different during EM retrieval from generation of ESF. In addition, there will be a difference in neural activation due to source monitoring. These are only four examples of testable predictions that follow from our framework; future work will likely reveal further and perhaps better examples.

In conclusion, the scenario construction framework and distinguishing between its three key components (EM traces, semantic information and scenario construction) puts us in a unique position to suggest novel accounts for several controversially discussed topics in EM research. In particular, I have argued that even though the mechanisms and functions of EM might overlap with other constructs such as SM and MTT into the future, EM is clearly distinct from these other constructs.

## Data Availability

This article has no additional data.

## Footnotes

^1^
‘Semantic’ is used differently in linguistics and philosophy and refers to the meaning of words [20]. Even in psychology, semantic memory is often used to refer to verbal content (e.g. [1]).

^2^
Tulving [26] introduced autonoetic consciousness to refer to the awareness that one has experienced a retrieved memory. By contrast, noetic consciousness refers to explicit awareness of possessing knowledge.

^3^
Many authors use the term ‘gist’ differently. Some use gist to describe the level of detail in the contents of EM, as in detail versus gist [49–52]. In our framework, this distinction is captured by the level of abstraction. Other authors use gist to mean ‘schematic’ and ascribe it to semantic memory that has been abstracted from EM [53,54].

^4^
‘Memory trace’ is used differently by many authors. Often it encompasses all mnemonic information stored by the brain, including neocortex (e.g. [20,59]). William James first introduced this concept and referred to it as ‘path of association’ [60].

^5^
Here and in the remainder of this article, I prefer the term ‘semantic information’ to ‘semantic memory’ to emphasize that semantic information is heterogeneous and is not likely stored in a single memory system and the retrieval of semantic information mostly does not intuitively appear as memory recall [40].

^6^
The content of a novel ESF can be encoded into EM and this memory item can be retrieved later, but the original ESF was not stored and retrieved, since, by definition, it was a novel simulation.
